# Effectiveness of non-pharmacological interventions for insomnia in children with Autism Spectrum Disorder: A systematic review and meta-analysis

**DOI:** 10.1371/journal.pone.0221428

**Published:** 2019-08-22

**Authors:** Sophie Keogh, Christopher Bridle, Niroshan A. Siriwardena, Amulya Nadkarni, Despina Laparidou, Simon J. Durrant, Niko Kargas, Graham R. Law, Ffion Curtis

**Affiliations:** 1 Lincolnshire Partnership Foundation Trust, Lincoln, United Kingdom; 2 Lincoln Institute for Health, University of Lincoln, Lincoln, United Kingdom; TNO, NETHERLANDS

## Abstract

**Background:**

Autism spectrum disorders (ASD) are a set of neurodevelopmental disorders characterised by behavioural, communication and social impairments. The prevalence of sleep disturbances in children with ASD is 40–80%, with significant effects on quality of life for the children and carers.

This systematic review aimed to synthesise evidence of the effects of behavioural interventions to improve sleep among children with ASD.

**Methods:**

Databases (MEDLINE, PsycINFO, CINAHL, ScienceDirect, Autism Data, CENTRAL, ClinicalTrials.gov and Current Controlled Trials) were searched for published, unpublished and ongoing randomised controlled trials evaluating the effect of non-pharmacological interventions for insomnia in children with autism spectrum conditions.

**Results:**

Three studies met the inclusion criteria, one provided actigraphy data, one Children’s Sleep Habits Questionnaire (CSHQ) data, and one both actigraphy and CSHQ data for use in meta-analyses. There were significant differences between the behavioural intervention and comparison groups (actigraphy data) for total sleep time (24.41 minutes, 95% CI 5.71, 43.11, P = 0.01), sleep latency (-18.31 minutes, 95% CI -30.84, -5.77, P = 0.004) and sleep efficiency (5.59%, 95% CI 0.87, 10.31, P = 0.02). There was also a favourable intervention effect evident for the subjective CSHQ data (-4.71, 95% CI -6.70, -2.73, P<0.00001). Risk of bias was low across several key domains (randomisation, allocation concealment and reporting), with some studies being unclear due to poor reporting.

**Conclusions:**

There are very few high quality randomised controlled trials in this area. Here we provide initial synthesised quantitative evidence of the effectiveness of behavioural interventions for treating sleep problems in children with ASD.

**Trial registration:**

Protocol was registered (CRD42017081784) on the International Prospective Register of Systematic Reviews (http://www.crd.york.ac.uk/PROSPERO).

## Background

Autism spectrum disorders (ASD) are a set of neurodevelopmental disorders characterised by behavioural, communication and social impairments [1]. Worldwide it is estimated that 1 in 160 children has an ASD [2]. Disordered sleep is one of the most common reasons parents seek medical support [3], with 40–80% of children with ASD having sleep difficulties [4]. Behaviours associated with disordered sleep in ASD include difficulty self-settling, frequent night waking, greater sleep onset latency, early waking and poor sleep efficiency (ratio of total sleep time to total time spent in bed) [4]. Disordered sleep can exacerbate some of the symptoms of ASD, such as over activity, disruptive behaviour, communication difficulties, repetitive behaviours and social skill deficits, which is not only impairing for the child, but will also increase family/caregiver stress [5, 6]. There are also lasting effects as adequate quantity and quality of sleep during childhood years is essential for neuronal development, which determines long-term trajectories of behaviour, learning, health and wellbeing [7].

In 2017, Cuomo et al. [7] conducted a meta-synthesis of eight reviews examining the efficacy of various sleep interventions, including pharmacologic treatments, alternative therapies and behavioural interventions, in children with ASD. Two of the included reviews [1, 8] focused on behavioural interventions in children with ASD, identifying a total of 15 studies. Overall Cuomo et al. [7] concluded that parent education and behavioural interventions showed the most promise for the treatment of multiple sleep problem domains. More recently, Kilpatrick et al. [9] conducted a systematic review of the literature focusing on the efficacy of parent training incorporated in behavioural sleep interventions for children with ASD and/or intellectual disabilities. They concluded that the inclusion of parent training within behavioural sleep interventions was considered generally effective, was valued by parents, and 9 of the 11 studies reported a reduction in sleep problems. These reviews included a broad range of studies (different research designs, diverse sample populations, a variety of intervention types, and a range of outcome measures), so the review authors [7, 9] were able to maximize the use of the evidence available; however, this was also limiting, as it was not possible to then statistically quantify intervention effects (e.g. a meta-analysis).

Sleep research is a rapidly developing area with emerging evidence indicating that sleep disturbances are not merely consequences of disease, but play important roles in their development [10]. Improving sleep outcomes is considered a priority in children with ASD, where development is already compromised [11]. The evidence for pharmacological interventions such as melatonin is inconclusive, reporting mixed findings in relation to dose, effectiveness and side-effects [12]. The National Institute for Health and Care Excellence [13] recommend that behavioural interventions are first line treatment for insomnia in ASD. Pharmacological interventions are only to be considered if sleep problems persist despite following the sleep plan (behavioural intervention), and are still only to be prescribed in conjunction with non-pharmacological interventions. Importantly, parents consider behavioural interventions as preferable to, and as effective as, medication [14]. Consequently, there is growing interest in the development of effective non-pharmacological interventions for treating insomnia. This is of particular importance in children with ASD and so supports the need to confirm the effectiveness of behavioural interventions in this population.

The aim of this review was to provide an up-to-date synthesis of the available evidence, focusing on behavioural interventions targeting sleep problems, compared to no intervention, in children with ASD.

## Methods

The inclusion criteria and methods of analysis were specified in advance and documented in a protocol that was registered (CRD42017081784) on the International Prospective Register of Systematic Reviews (http://www.crd.york.ac.uk/PROSPERO). This review was reported in accordance with the Preferred Reporting Items for Systematic review and Meta-Analysis’ (PRISMA) guidelines [15] (S1 PRISMA Checklist).

### Eligibility criteria

Studies were considered for inclusion if they were randomised controlled trials (RCTs) of sleep-based behavioural interventions for children with ASD. In order for a study to be considered for inclusion the sample would have to be children aged 18 years or under. The review included studies in which participant eligibility required a pre-existing diagnosis of ASD. Studies of behavioural interventions targeting sleep compared with concurrent control that reported relevant sleep outcome measures were eligible. Behavioural interventions were defined as an intervention which uses behavioural techniques, such as reinforcement to support the desired behaviour.

### Study identification

To identify existing relevant systematic reviews, published, unpublished and ongoing trials, the following electronic databases were searched from inception to January 2019: MEDLINE, PsycINFO, CINAHL, ScienceDirect, Web of Science, Autism Data, CENTRAL, ClinicalTrials.gov and Current Controlled Trials. No restrictions on language were imposed. Database searching was supplemented with internet searching (e.g. Google Scholar), and forward and backward citation tracking from systematic reviews and included studies. Key search terms for database searching included the following:

*("Autism spectrum disorder*" *OR asd OR autism) AND (sleep OR insomnia OR "bedtime resistance" OR dyssomnia) AND (child OR toddler OR infant OR adolescent OR preschool OR teenage OR pediatric OR paediatric OR childhood)*

Search terms used in the MEDLINE search are provided in Supplementary Material: S1 Electronic Search Strategy.

Search results were downloaded to Endnote, and duplicate citations were removed. Two reviewers independently screened titles and abstracts against the inclusion criteria. Where studies could not be excluded based on title and abstract, two reviewers independently assessed full papers for relevance. Any discrepancies were resolved through discussion or, where required, through involvement of a third reviewer.

### Data abstraction

Data were extracted by one reviewer and checked for accuracy by another using a template that included: (a) study details, for example title, aim, design and type of intervention; (b) participant information, for example age, gender, diagnosis, severity, co-morbidities and inclusion/exclusion criteria; (c) groups, for example number randomised, description of intervention and comparator, delivery, content, frequency, duration and provider; (d) outcomes, for example primary outcome name, definition, type, how it is measured and reported, missing data and reasons for missing data.

### Risk of bias assessment

Two reviewers independently assessed risk of bias (Cochrane Risk of Bias assessment tool) on key dimensions including: Random sequence generation, allocation concealment, blinding, and incomplete outcome data. Each domain was classified as adequate (low risk of bias), inadequate (high risk of bias) or unclear (not possible to determine risk of bias). An overall study risk of bias was not assessed and data for each domain are presented for readers to interpret in context with review findings. Risk of bias assessment was not used as a reason for exclusion.

### Data analysis

All analyses were conducted using Review Manager (RevMan) version 5.3 [16] software for Windows. All studies reported changes in sleep as a continuous outcome. The primary outcome was a mean difference in total sleep duration (actigraphy data), which was reported in 3 studies [17]. Secondary outcomes included sleep latency and sleep efficiency (actigraphy data), and total score from the CSHQ [18]. The summary measure of treatment effect for both primary and secondary outcomes was the between groups difference in sleep outcomes, expressed as mean difference (MD).

Random-effects models were used in all meta-analyses, as they are more conservative than the fixed effects models since, by incorporating within- and between-study variance, the confidence intervals for the summary effect are wider. Statistical heterogeneity was assessed using the I^2^ test, which described the percentage of variability among effect estimates beyond that expected by chance. In cases where heterogeneity was considered to be important (I^2^ values ≥40%), sources of clinical and methodological diversity were explored.

## Results

**Fig 1:**
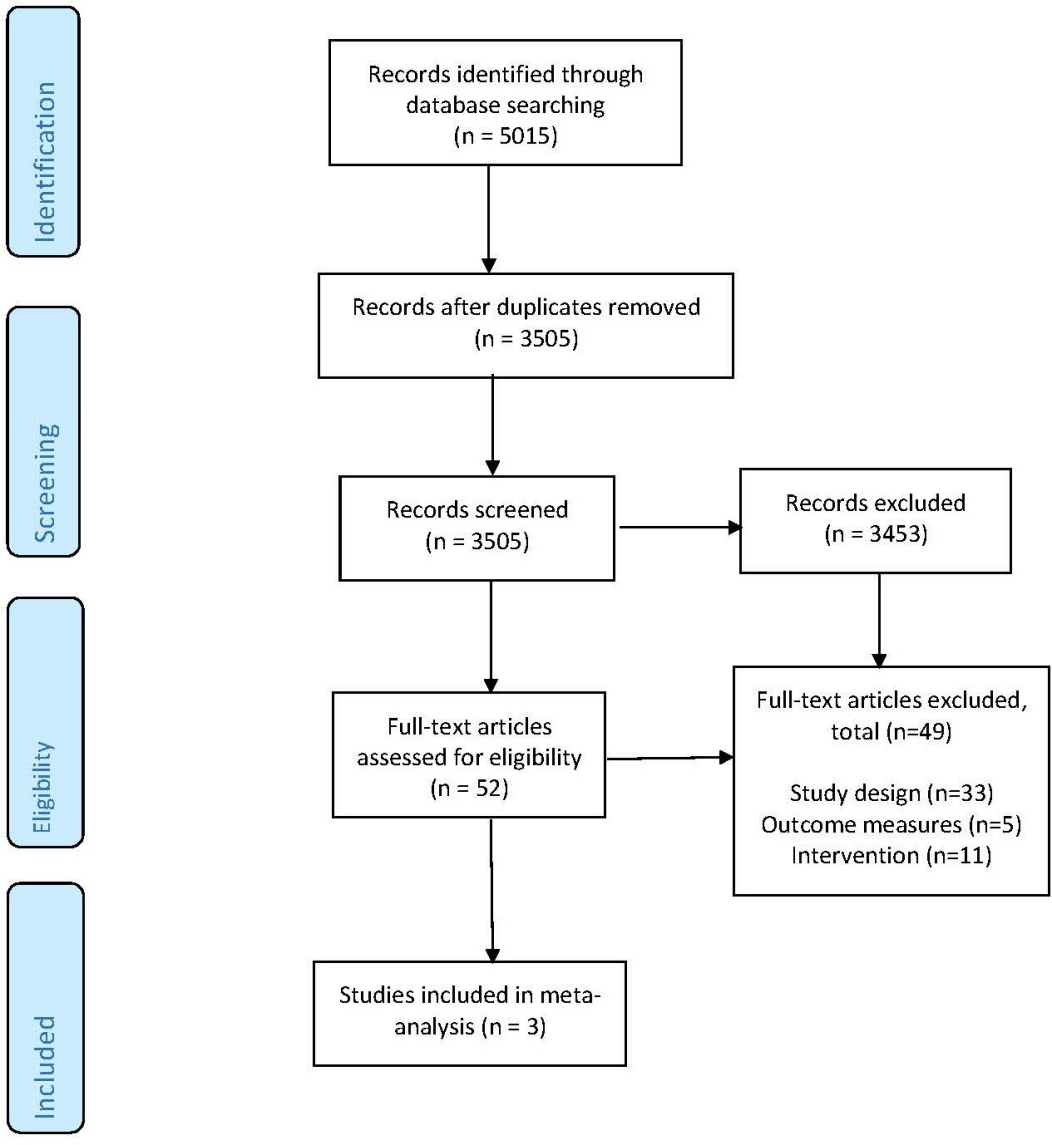
Flow diagram of study selection [15].

After removal of duplicate citations, the search strategy identified 3,505 distinct citations, of which 3,453 were excluded during the initial screening phase (Fig 1). For the remaining 52 citations, full text papers were ordered. Three studies met the inclusion criteria [17, 19, 20]. The main reasons for exclusion of full text papers were study design (n = 33), outcome measures (n = 5), and intervention (n = 11).

### Characteristics of included studies

The three included studies [17, 19, 20] were published between 2012 and 2015, see Table 1. These were conducted in the US [17], Australia [20] and Italy [19]. The three studies randomised 146 participants, with intervention group sample sizes ranging between 18 and 33 participants. The mean age of study populations ranged from 6.3 years [17] to 10.3 years [20].

**Table 1:** Study characteristics.

Study (location)	Sample size, age (years), sex	Intervention, frequency, duration and components.	Comparison	Baseline data: mean±SD, end-time point
**Cortesi et al., (2012)** **Rome [19]**	Intervention: *n* 33 Age: 7.1 (0.7) 83% male Control: *n* 32 Age 6.3 (1.2) 84% male	^1^Four-arm study. Baseline evaluation and 4-weekly 50 minute CBT sessions over 12 weeks to include a sleep focused multifactorial intervention with cognitive, behavioural and educational components. Session 1: information giving about sleep (nature, function and regulation), Session 2: parents were instructed to create consistent sleep schedule, session 3: reviewing instructions and identifying individual difficulties and implementation of methods to replace any remaining maladaptive behaviours with more appropriate sleep habits, session 4: reviewed instructions and information on prevention of insomnia relapse. Additionally, parents attended twice-monthly individually tailored CBT sessions. The focus of maintenance sessions was on consolidating treatment strategies learned during initial therapy and developing methods for coping for residual insomnia.	Placebo provided during fortnightly 15 minute meetings to match melatonin arm of study.	**Actigraphy**^**2**^ **TST** Intervention: 408.08 (49.03) Control: 413.00 (45.13) **Sleep onset delay** Intervention: 76.34 (31.70) Control: 78.20 (33.83) **Sleep efficiency %** Intervention: 71.37 (4.77) Control: 71.13 (4.99) **CSHQ** Intervention: 64.48 (5.48) Control: 64.20 (4.85), 12 weeks
**Adkins et al., (2012)** **North America [17]**	Total age 6.4 ±6 2.6 Intervention: *n* 18 age 55% male Control: *n* 18 Age 78% male	Sleep education pamphlet, self-administered, (2 weeks) to include (1) providing a comfortable sleep setting; (2) establishing regular bedtime habits;(3) keeping a regular schedule; (4) teaching your child to fall asleep alone; (5) avoiding naps (in children who have outgrown the need for a daytime nap); and (6) encouraging daytime activities that promote a better sleep/wake schedule.	No pamphlet. Control group to receive pamphlet after the study was completed.	**Actigraphy**^**2**^ **TST** Intervention: 465.7 (66.3) Control: 461.4 (42.4) **Sleep onset delay** Intervention: 56.7 (27.1) Control: 52.1 (25.1) **Sleep efficiency %** Intervention: 75.5 (6.1) Control: 76.8 (6.0), 2 weeks
**Papadopoulos et al., (2015)** **Australia [20]**	Intervention: *n* 28 Age 10.3 (1.7) 85.7% male Control: *n* 33 Age 9.8 (2.0) 90.9% male	Two face-to-face sleep consultations and follow-up phone call with clinician 2 weeks apart. An assessment of child’s sleep problem, discussion of parental goals, and psychoeducation about normal sleep, sleep cycles, and sleep hygiene strategies. During this consultation, a tailored behavioural sleep management plan was formulated. The second consultation and follow-up phone call were used to review the sleep diary, reinforce strategies, and troubleshoot any problems. Information sheets addressing normal sleep, common sleep problems, and strategies for managing specific problems were provided.	Usual care (for ADHD): Paediatrician apt typically every 6 months to check height, weight, and blood pressure and to re-issue a prescription medication and/or brief behavioural strategies which do not routinely include sleep strategies.	**CSHQ** Intervention 56.7 (6.6) Control 57.7 (9.4), 3 months

^1^four-arm trial: combination therapy group, melatonin group, CBT group and placebo group.

^2^Actigraphy TST: total sleep time

Participants within the included studies were all required to have a diagnosis of ASD. In two [17, 19] of the three studies, diagnosis was based on clinical evaluation using the Diagnostic Statistical Manual–Fourth Edition-TR (DSM-IV-TR, American Psychiatric Association 2000) and confirmed by the Autistic Diagnostic Interview–Revised (ADI-R) and/or the Autism Diagnostic Observation Schedule–generic (ADOS-G). In the third study, a clinician diagnosis of an ASD was confirmed by parents of the participants [20].

To identify sleep problems in participating children, two studies [17, 19] used parental report of sleep difficulties, including consideration of sleep onset latency, wake after sleep onset or night-time awakenings. The third study required participants to have sleep onset disorder, limit setting disorder, delayed sleep phase or insomnia as defined by the American Academy of Sleep Medicine [20].

Common participant exclusion criteria in the individual studies included current receipt of pharmacological treatments, psychotherapy or a behavioural intervention, sleep disordered breathing [19], Periodic Limb Movement [19], serious medical or mental health conditions [20], and sleep apnea [17, 20].

Delivery of behavioural interventions varied across the different studies. Adkins et al. [17] provided an education pamphlet for parents to read and follow without further instruction from the study staff. Experienced clinical psychologists delivered four-weekly 50-minute face-to-face CBT sessions in the Cortesi et al. [19] study. This was delivered in an outpatient university clinic and completion was defined as completion of the baseline session and at least two treatment sessions. Papadopoulos et al. [20] used two face-to-face sleep consultations and a follow-up phone call with a trained clinician, each two weeks apart, to deliver their intervention. This study also provided the most details in relation to sleep disorders, definitions and examples of behavioural interventions (e.g. sleep onset association disorder, child associates falling asleep with a certain objector person which would be addressed by Parental presence at sleep time being managed with adult fading/graduated extinction).

All studies were two-arm design RCTs (intervention vs control) with the exception of Cortesi et al. [19], who investigated the relative and combined effects of melatonin and CBT, compared to a placebo control (four-arm). Primary outcomes for the included studies were sleep measures; actigraphy data [17], CSHQ scores [20] or both actigraphy data and CSHQ scores [19].

Of the 28 participants in the intervention group of the Papadopoulos et al. [20] study, 26 were taking medications such as Ritalin, Concerta, or Clonidine. In the control group, 27 of the 33 participants were taking medications: Ritalin, Concerta, and Atomexetine. Five children in the intervention group (n = 18) in the Adkins et al. [17] study were taking psychotropic medications, three were taking melatonin and two stimulants. Within the control group (n = 18), nine were taking psychotropic medications, three melatonin and two stimulants. All children in the Cortesi et al. [19] study were drug free for at least six months and no children receiving psychotherapy or behavioural interventions were enrolled in the study.

**Fig 2:**
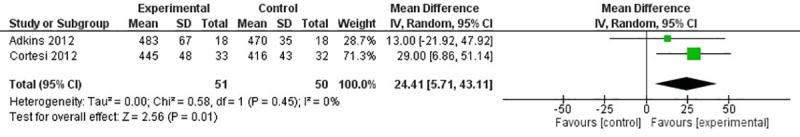
Actigraphy total sleep time (minutes) in children with ASD.

**Fig 3:**
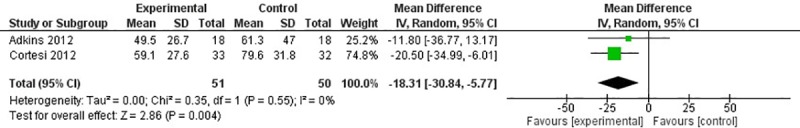
Actigraphy sleep onset latency (minutes) in children with ASD.

**Fig 4:**
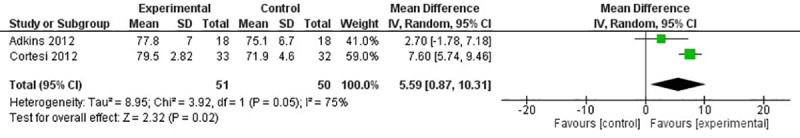
Actigraphy sleep efficiency (%) in children with ASD.

Of the two studies [17, 19] that used actigraphy devices to measure sleep outcomes, data for total sleep time measured in minutes (Fig 2), sleep onset latency (time from lights out to measured first sleep onset) (Fig 3), and sleep efficiency (ratio of total sleep time to total time in bed) (Fig 4) were available for statistical analysis. Adkins et al. [17] collected actigraphy data via AW Spectrum Acti-watch devices (Phillips Respironics, Bend, OR) for two weeks following a 2-week pamphlet intervention period. Cortesi et al. [19] monitored each child with an actigraphy device in the zero crossing mode from the Ambulatory Monitoring Inc. (Ardsley, NY, USA) for a minimum of seven nights following 12 weeks of CBT. Actigraphy devices were used in conjunction with parent report sleep diaries in both of the studies. Adkins et al. [17] used the devices to confirm parent report and Cortesi et al. [19] recommended parents kept diaries to enable editing of actigraphy data for potential artefacts and failures.

**Fig 5:**
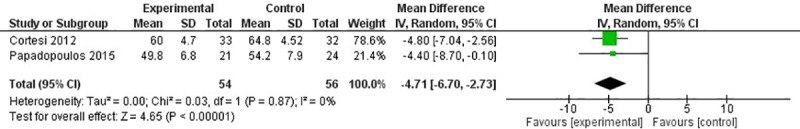
Children’s sleep habits questionnaire total score.

Two studies [19, 20] reported outcome data using the CSHQ. This is a standardised questionnaire designed for use in children. It includes 45 items and is rated retrospectively over the previous week to screen for common sleep problems in key areas such as child bedtime, sleep behaviour and night time waking. Both studies reported a total sleep disturbance score for the questionnaire, which was used for the pooled data synthesis (Fig 5). A total score of 41 is considered the optimal clinical cut-off for sleep disturbance [18].

Meta-analysis of two studies [17, 19] demonstrated a statistically significant increase in total sleep time following behavioural sleep interventions (Fig 2, 24.41, 95% CI 5.71, 43.11, P = 0.01). There was no indication of statistical heterogeneity among the studies reporting total sleep time (I^2^ = 0%). The synthesised data from these two studies also demonstrated a significant between groups mean difference (-18.31, 95% CI -30.84, -5.77, P = 0.004) in sleep onset latency, favouring the intervention group (Fig 3). When sleep efficiency (%) data from the two studies were synthesised, a statistically significant effect was observed on sleep efficiency following a behavioural sleep intervention (5.59, 95% CI 0.87, 10.31, P = 0.02); however, substantial heterogeneity (Chi^2^ = 3.92, df = 1, P = value = 0.05; I^2^ = 75%) was evident (Fig 4). Data are presented here as a sensitivity analysis was not possible with only two studies included. The final meta-analysis synthesising CSHQ data from two studies [19, 20] demonstrated a statistically significant behavioural intervention effect when compared to control (-4.71, 95% CI -6.70, -2.73, P<0.00001), with no evidence of statistical heterogeneity (I^2^ = 0%) (Fig 5).

### Risk of bias

Randomisation processes were well described in all studies and as such random sequence generation risk of bias was low for all studies (see Table 2). Allocation concealment was considered to be of a low risk of bias for two studies [19, 20] and unclear in the remaining study [17] due to absence of reporting. It was not possible to blind participants to which arm of the intervention they had been randomised to, hence, all studies were considered to be high risk of bias for the category ‘blinding of participants and personnel’. The assessment of risk of bias for the domain ‘blinding of outcome assessment’ is presented separately for parent-reported CSHQ and the more objective actigraphy measure. The actigraphy data from the Adkins et al. [17] study was uploaded to a database for centralized scoring, by a single individual who had no contact with participants. There was no information available for the Cortesi et al. [19] study. It was considered that there could be a higher risk of bias for the CSHQ, as parents were the first line assessors, subjectively scoring their children’s sleep behaviours. Again data processing procedures were not described. The risk of bias for ‘Incomplete outcome data’ was considered high in the Papadopoulos et al. [20] study, with only 75% of intervention group and 73% of usual care group providing follow up data. All studies were considered to have low risk of bias for ‘selective outcome reporting’ as all measures described were reported in the results section.

**Table 2:** Risk of bias.

Study ID	Random sequence generation	Allocation concealment	Blinding of participants and personnel	Blinding of outcome assessment (Actigraphy)	Blinding of outcome assessment (CSHQ)	Incomplete outcome data	Selective outcome reporting
**Papadopoulos 2015 [20]**	low	low	high	-	high	high	low
**Cortesi 2012 [19]**	low	low	high	Unclear	high	low	low
**Adkins 2012 [17]**	low	unclear	high	Low	-	low	low

## Discussion

The aim of this review was to systematically review and quantify the effects of behavioural interventions targeting sleep problems in children with ASD. Three studies evaluating the effect of behavioural interventions on sleep outcomes in children with ASD were retrieved. One of the studies [17] provided actigraphy data, one total scores for CSHQs [20], and one study [19] reported both actigraphy and CSHQ data.

Synthesised actigraphy data showed a statistically significant effect of behavioural interventions compared to control on children’s total sleep time (24 minutes), sleep onset latency (-18 minutes) and sleep efficiency (5.5%). Substantial heterogeneity was evident in the pooled analysis of the sleep efficiency data; the reason for this was not clear, so data were still presented. The final meta-analysis combining CSHQ total scores from two studies were in agreement with the actigraphy results, demonstrating a significant intervention effect (-4.7 CSHQ score) when compared to non-intervention comparison groups.

### Strengths and weaknesses of study

To inform the methodology, selection, evaluation and syntheses of relevant evidence, this study adhered to the PRISMA reporting guidelines and a rigorous pre-specified protocol (PROSPERO), thus, limiting the potential for bias. Comprehensive searching of multiple electronic databases for published and unpublished work, bibliography scanning and contacting authors identified only three published studies [17, 19, 20], all of which had relatively small sample sizes. Despite this, data were available to conduct the first meta-analysis, synthesising the effects of behavioural interventions targeting sleep problems in children with ASD.

A potential weakness of this review was the absence of data from unpublished studies, which could increase the potential risk of publication bias. Due to the small number of studies included within this review, it was not possible to conduct a funnel plot. However, publication bias was deemed unlikely, as two of the three included studies did not report significant effects for the outcomes of interest in this review.

Evidence from individual studies in the current review was synthesised despite variation in intervention delivery (face to face, pamphlet) and duration (two to 12 weeks). There are only a limited number of studies in the area and the decision to combine data from the included studies was based on previous research that observed similar positive treatment outcomes from different types of interventions (group vs individual interventions and those ranging from two to five weeks) [9].

Both objective and subjective outcome measures were included within this review, as they provide complementary information [21]. There was some variation in placement of actigraphy devices in the Adkins et al. [17] study, where wrist placement was tolerated by 27 (75%) of the children, whereas nine (25%) children required the shoulder placement. There were no differences reported for the actigraphy data in children who wore actigraphy devices on the wrist or shoulder [17]. Actigraphy devices are now commonly used in sleep research [22] for the assessment of various sleep parameters, including total sleep duration, sleep onset latency and sleep efficiency. Future work should explore variation in actigraphy device placement in children where wrist placement may not always be well tolerated.

Subjective measures, such as the CSHQ, are important as they reflect the subjective perception, which is arguably more ‘ecologically’ sound and is crucial in the management process of the disorder [21]. The CSHQ demonstrates adequate internal consistency and acceptable test-retest reliability in typically developing children [18] and is currently the most widely used standardized sleep assessment tool for children with ASD [22].

### Discussion of findings

The interventions in the included studies aimed to educate parents with strategies to improve sleep behaviours in their children, which is considered the best evidence-based practice in this population [23]. The depth of descriptions of the interventions included within the primary papers varied, with Papadopoulos et al. [20] providing the most information. Components of the behavioural intervention described by Papadopoulos et al. [20], such as emphasizing a calming bedtime using relaxation training, would be considered effective for many forms of insomnia, including those related to core deficits of ASD [24]. Cortesi et al. [19] provided an outline of each of the sessions. The Adkins et al. [17] study reported six areas relevant to promoting sleep among children with ASD provided within the pamphlet in the Adkins et al. [17] study. It was, however, clear from the information available that the interventions were all quite different from one another with regards to content and ‘dose’, which should be explored further.

The summary effect for total sleep duration and sleep onset delay demonstrated statistically significant differences favouring the intervention groups. Only one [19] of the included original studies reported a significant effect post-intervention. This study had the largest sample size and was the only included study in which children were free from medication. Despite children in the two smaller studies [17, 20] being on one or more medications, a promising treatment effect was still observed post intervention. In addition to establishing the effectiveness of behavioural interventions for insomnia in a drug free population, future research should explore this in the presence of medications, as it is estimated that 30%–65% of children with ASD use at least one psychotropic medication [25].

Both of the included actigraphy studies [17, 19] reported a significant intervention effect for sleep efficiency. The improvement in the original pamphlet study [17] was only a two percentage point absolute change, from 75% to 77%, which is unlikely to be clinically meaningful. This was larger in the Cortesi et al. [19] study (8%) and the between groups difference presented here was 5.5%, which may be clinically meaningful for some participants. Different delivery formats warrant further investigation as, in agreement with Kilpatrick [9], this review indicates that both face-to-face CBT and a more simple information pamphlet are effective in this population. The individual point estimate from the meta-analysis presented here indicated a positive post intervention effect for the Adkins et al. [17] study, which was only a two week self-administered pamphlet.

The CSHQ total score between-groups difference favoured the intervention and was statistically significant. It is difficult to determine if this is clinically meaningful, as the CSHQ was developed to classify sleep (e.g. a total score of 41 is considered the optimal clinical cut-off for sleep disturbance; [18]) and as such should be used in conjunction with other sleep measures when evaluating interventions.

Risk of bias was low across several key domains (randomisation, allocation concealment and reporting) with some studies being unclear due to poor reporting. There were some domains where it was difficult to eliminate potential for bias due to study design; for example, blinding of participants and personnel. Whilst it is unlikely this would directly influence the children’s actigraphy data, risk of bias may influence parents’ reporting for the CSHQs and diaries that were kept in conjunction with the actigraphy data. In addition to this, parents would not have been blinded to outcome assessment when completing CSHQs.

Previous reviews [1, 7–9] have included a broader range of studies to provide a comprehensive overview. However, by including studies with different designs, intervention types and participant characteristics, it has not been possible to draw definitive conclusions quantifying intervention effects. A recent review [26] suggests a transdiagnostic treatment approach across different developmental orders may be effective; however, they conclude more research is needed prior to any recommendations being made. In comparison, this review had a narrow focus to begin refining the evidence in this area by statistically quantifying the effects of behavioural interventions in children with ASD. Future research should also consider focusing on children with ASD and not grouping them with other ‘developmental disorders’ as previous studies (blindness, Down syndrome in [27]) have done, as children with different conditions would likely respond differently to behavioural interventions. This is particularly relevant when considering sleep problem behaviours in children with ASD.

### Implications

The evidence presented in the current review will be useful for clinical practice in the management of sleep problems in children with ASD, enabling clinicians and families to make informed choices. Despite behavioural interventions being the recommended first line treatment, and sleep medication being associated with lower quality-of-life scales and more challenging daytime behaviours, pharmacological prescribing is common with many children being prescribed more than one type of sleep medication [25]. Behavioural interventions are advantageous as they are preferred by parents and have positive effects on sleep difficulties in ASD without the risk of adverse effects associated with pharmacological approaches [12]. Behavioural interventions also have the additional advantage that they can be delivered for long periods of time if required, and in case of relapse, without concerns about untoward effects associated with long term use of medication.

Here we provide preliminary quantitative evidence of the effectiveness of behavioural interventions for treating sleep problems in children with ASD. Future research should explore the effect of behavioural interventions alone and in combination with other medications in children with ASD.

In preparing this review it was evident that interventions targeting sleep problems is a rapidly evolving area of research. This review has presented all available evidence to date, however, the authors are aware of at least one ongoing study with a planned completion date of 2023; Telehealth Delivery of Treatment for Sleep Disturbances in Young Children With Autism Spectrum Disorder [28].

## Supporting information

S1 PRISMA Checklist (DOC)

S1 Electronic Search Strategy (DOCX)
