# Supplementary material for: Effectiveness of non-pharmacological interventions for insomnia in children with Autism Spectrum Disorder: A systematic review and meta-analysis
Source: PLoS One. 2019 Aug 22;14(8):e0221428. doi: 10.1371/journal.pone.0221428 (PMC6705823; doi:10.1371/journal.pone.0221428)
Supplement: S1 Electronic Search Strategy — (DOCX) [file pone.0221428.s002.docx]

MEDLINE via EBSCO: full electronic search strategy (from inception to January 2019)

1. mh “Autism Spectrum Disorder”
2. Autis* spectrum disorder
3. Autis*
4. mh “Autistic Disorder”
5. ASD
6. Autis* spectrum condition
7. ASC
8. mh “Asperger Syndrome”
9. Asperger Syndrome
10. Asperger
11. Neurodevelopmental disorder
12. Neuro-developmental disorder
13. 1 OR 2 OR 3 OR 4 OR 5 OR 6 OR 7 OR 8 OR 9 OR 10 OR 11 OR 12
14. mh “Child”
15. Child*
16. Toddler
17. Adolescen*
18. mh “Adolescent”
19. Teenage*
20. mh “Infant”
21. Infant
22. Paediatrics
23. mh “Pediatrics”
24. Pediatrics
25. mh “Child, Preschool”
26. Preschool (TX)
27. 14 OR 15 OR 16 OR 17 OR 18 OR 19 OR 20 OR 21 OR 22 OR 23 OR 24 OR 25 OR 26
28. Mh “Sleep”
29. Sleep
30. mh “Sleep Wake Disorders”
31. Sleep wake disorders
32. Sleep disorders
33. Sleep problems
34. Sleep disturbance
35. mh “Sleep initiation and maintenance disorders”
36. Sleep initiation and maintenance disorders
37. Insomnia
38. mh “Dyssomnias”
39. Dyssomnias
40. Bedtime resistance
41. 28 OR 29 OR 30 OR 31 OR 32 OR 33 OR 34 OR 35 OR 36 OR 37 OR 38 OR 39 OR 40
42. 13 AND 27 AND 41
